# Using Relational Agents to Promote Family Communication Around Type 1 Diabetes Self-Management in the Diabetes Family Teamwork Online Intervention: Longitudinal Pilot Study

**DOI:** 10.2196/15318

**Published:** 2019-09-13

**Authors:** Debbe Thompson, Chishinga Callender, Caroline Gonynor, Karen W Cullen, Maria J Redondo, Ashley Butler, Barbara J Anderson

**Affiliations:** 1 United States Department of Agriculture/Agricultural Research Service Children's Nutrition Research Center Baylor College of Medicine Houston, TX United States; 2 Department of Pediatrics Baylor College of Medicine Houston, TX United States; 3 Diabetes and Endocrinology Section Department of Pediatrics Baylor College of Medicine Houston, TX United States

**Keywords:** adolescents, family communication, preadolescents, relational agent, type 1 diabetes

## Abstract

**Background:**

Family conflict can reduce adolescent adherence to type 1 diabetes management tasks. The Family Teamwork in-person intervention was shown to be efficacious in reducing conflict and low adherence to diabetes-related tasks. Its reach and potential impact, however, were limited by the need to deliver the intervention sessions in person. Relational agents (ie, computerized versions of humans) have been shown to appeal to diverse audiences and may be an acceptable replacement for a human in technology-based behavior change interventions.

**Objective:**

The purpose of this paper is to present the results of a pilot study assessing feasibility and acceptability of Diabetes Family Teamwork Online, an adapted version of the Family Teamwork intervention, delivered over the internet and guided by a relational agent.

**Methods:**

Parent-adolescent dyads were recruited through a diabetes care clinic at a large tertiary care hospital in the southwestern United States. A one-group design, with assessments at baseline, immediate postintervention, and 3 months later, was used to assess feasibility. A priori feasibility criteria included an assessment of recruitment, completion, attrition, program satisfaction, therapeutic alliance, attitudes toward the relational agent, and data collection. The institutional review board at Baylor College of Medicine approved the protocol (H-37245).

**Results:**

Twenty-seven adolescents aged 10 to 15 years with type 1 diabetes and their parents were enrolled. Criteria used to assess feasibility were (1) recruitment goals were met (n=20), (2) families completed ≥75% of the modules, (3) attrition rate was ≤10%, (4) program satisfaction was high (≥80% of families), (5) therapeutic alliance was high (average score of ≥60/84), (6) families expressed positive attitudes toward the relational agent (average item score of ≥5 on ≥4 items), (7) ≥80% of data were collected at post 1 and post 2, and (8) few technical issues (≤10%) occurred during intervention delivery. All feasibility criteria were met. Qualitative data confirmed that adolescents and parents had positive reactions to both the content and approach.

**Conclusions:**

The Diabetes Family Teamwork Online intervention proved to be a feasible and acceptable method for enhancing communication around diabetes management tasks in families with an adolescent who has type 1 diabetes.

**International Registered Report Identifier (IRRID):**

RR2-10.2196/resprot.5817

## Introduction

According to the most up-to-date statistics from the SEARCH for Diabetes in Youth Study Group [1,2], about 154,000 youth are living with type 1 diabetes (T1D), and each year approximately 15,000 youth aged 20 years and younger are diagnosed with T1D, which is second only to asthma as the most common chronic disease of childhood in the United States [3]. Among youth, from 2001 to 2015, 27,000 new cases were diagnosed annually, with children aged 10 to 14 years having the greatest incidence of T1D [4].

Results from the Diabetes Control and Complications Trial (DCCT) increased awareness of how important maintaining near-normal blood glucose levels is to the management of T1D, particularly for delaying and/or preventing T1D complications [5]. However, translation of the rigorous DCCT regimen has been limited by low patient adherence to treatment plans [6]. Adolescents are particularly at risk for low adherence to the constant demands associated with T1D disease management. An efficacy trial of continuous glucose monitoring systems (CGMSs) reported that the adult cohort, but not the adolescent cohort, improved blood glucose control using CGMSs. This may be partially explained by the significantly lower CGMS adherence in the adolescent cohort [7], emphasizing that technology alone, without impacting the environments in which adolescents live and manage T1D, may not produce improved blood glucose control.

It is well documented that the family is important in adolescent adherence [8]. Family conflict and negative communication around diabetes management, especially blood glucose monitoring, are common barriers to adolescent adherence to the diabetes regimen [9]. A meta-analysis of T1D interventions promoting adherence in youth concluded that behavioral interventions without a focus on the personal and interpersonal aspects of the disease were less likely to affect blood glucose control [10]. Therefore, interventions focusing on adolescent adherence and disease management should not ignore the importance of the family, particularly family communication around T1D management tasks, in this process [8].

The Family Teamwork (FT) intervention is an 8-session, in-person intervention delivered by a trained research assistant during routine clinic visits to youth aged 10 to 14 years with T1D and their parents. It was designed to improve hemoglobin A_1c_ (HbA_1c_) by promoting adherence and blood glucose control in adolescents with T1D [11]. Sessions focused on helping parents and adolescents work together as a team to increase positive parent involvement and reduce family conflict around T1D management. Its efficacy has been demonstrated in two randomized controlled trials, with significant improvements observed in blood glucose monitoring adherence, HbA_1c_ [12,13], and self-reported quality of life [9] in adolescents who participated in FT as compared with those who received standard care only. There was no increase in diabetes-related family conflict, and parents maintained or increased involvement in diabetes management tasks [9]. Reach, however, was limited by the costs associated with having a trained research assistant available to deliver the intervention, the number of families the intervention could be delivered to, and the need for families to travel to the clinic to participate in the intervention.

Communication technologies offer an opportunity to strengthen the reach and effectiveness [14] of interventions while reducing delivery cost. A review found effective eHealth technologies extended the reach of adult diabetes management interventions [15]. However, no studies of online self-management interventions for youth with T1D that met methodological criteria were identified [16], and most studies found lacked details on intervention components and how interventions were tailored for individual patients. An online program to meet the social and informational needs of older adolescents and young adults with T1D was found to be feasible, but only with multiple reminders from the clinic team [17]. Several small online interventions with youth with T1D improved self-management behavior [18,19]. One reported that translating an in-person coping skills intervention for adolescents with T1D that improved glycemic control to the internet was feasible [20].

Self-management is essential to successful treatment and control of chronic diseases like T1D [5,21]. A meta-analysis of pediatric eHealth interventions suggested that interventions with behavioral methods like self-monitoring, goal-setting, and problem-solving were much more effective than those that were solely educational [22], complementing the findings of the meta-analysis cited previously [10]. Both support that adherence and blood glucose control would be improved by intervening with the parent/adolescent dyad to address family barriers around adolescent adherence, particularly when supported by behavioral self-management (goal-setting, problem-solving, self-monitoring). The gold standard for health behavior change is face-to-face interaction with a health care provider [23,24]; however, limitations such as reach [24], time [23], cost, and fidelity can reduce effectiveness [23].

Relational agents (Ragts) may address these limitations. Ragts are computer representations of health care providers that simulate face-to-face interaction with an individual in the real world [23]. They mimic characteristics of face-to-face interactions, including verbal and nonverbal behaviors associated with trust-, rapport-, and relationship-building. Programs delivered by Ragts are convenient, readily accessible, and relatively low in cost, particularly when delivered online [23]. Ragts have been found to be feasible and acceptable with a variety of age groups and health behaviors [23,25-30]. The purpose of this paper is to report the feasibility of an intervention, Diabetes Family Teamwork Online (FTO), an updated version of FT adapted for online delivery by a Ragt.

## Methods

### Adaptation of Family Teamwork Into Diabetes Family Teamwork Online

Using an approach informed by the authors’ previous work in the development of technology-based interventions, computers as persuasive technologies [31], and self determination theory (SDT) [32], FT was systematically adapted for online delivery. Informed by social cognitive theory [33], FT contained 8 informational sessions and 1 review session focused on healthy family communication around key T1D management tasks and common T1D-related issues encountered by families, such as avoiding diabetes burnout (Table 1). A trained research assistant delivered the sessions to parent/adolescent dyads during routine visits to the adolescent’s diabetes care provider, meaning that an extended period of time likely elapsed between clinic visits (eg, 3 to 4 months). Therefore, by necessity, there was a fair amount of repetition included in the sessions. After reviewing FT session content, the research team determined that the sessions could be collapsed into 4 online modules delivered to families every 2 weeks without loss of fidelity to the original program.

**Figure 1:**
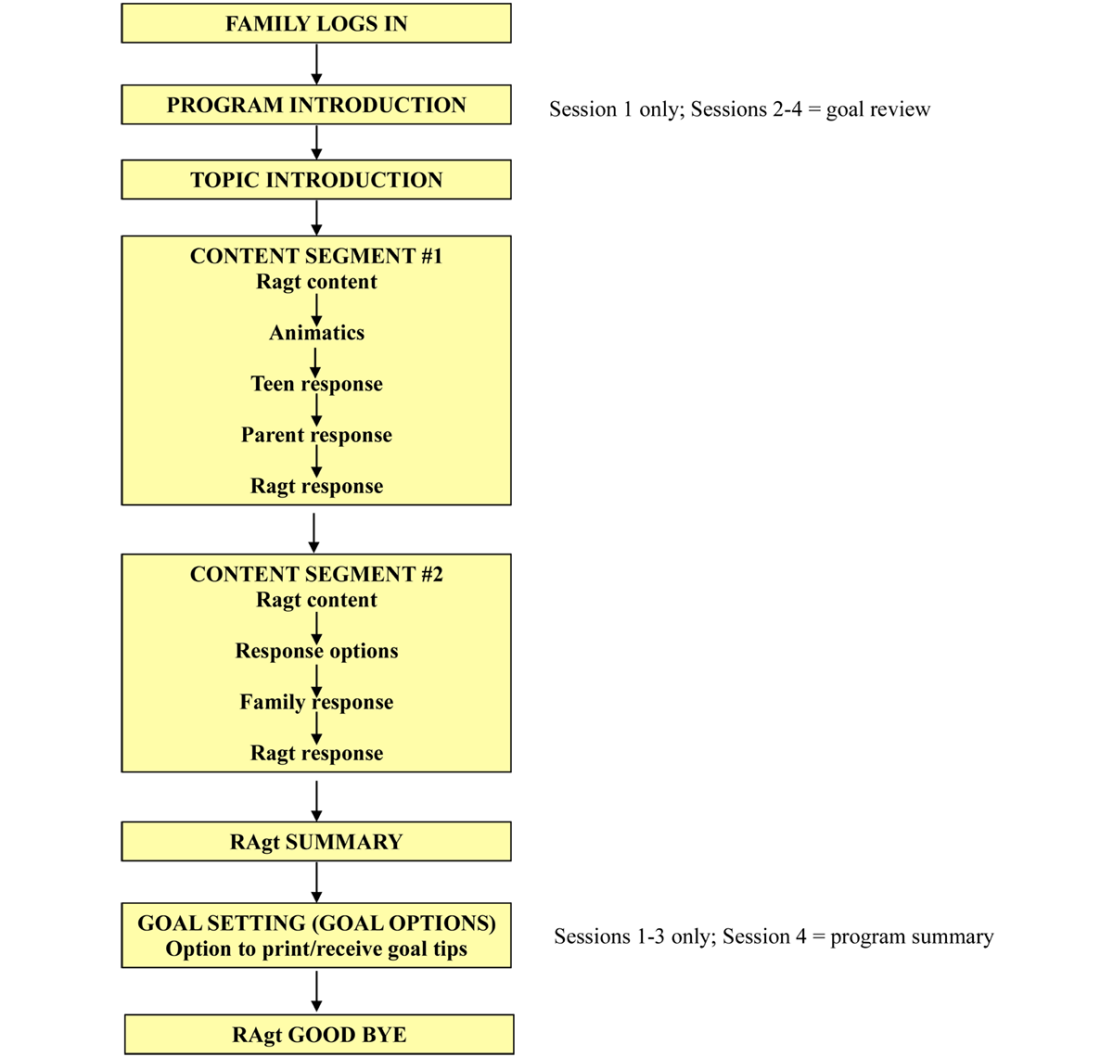
Family Teamwork Online module flow.

**Figure 2:**
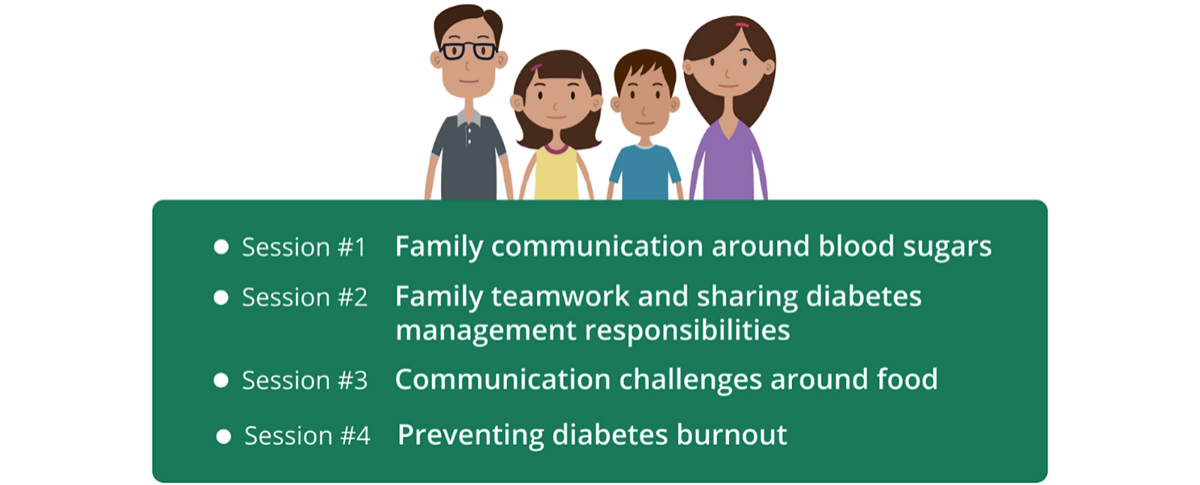
Family Teamwork Online module topics.

To facilitate translation and ensure a balance between didactic components and interactivity, a flow diagram was developed to guide the online program (Figure 1). Using feedback obtained from interviews with families during development [34], a team consisting of a key member of the FT development team (BJA), a pediatric endocrinologist (MJR), and a child psychologist (AB) collapsed the 8 sessions into 4 online modules (Figure 2). Using the flow diagram as a guide, scripts were written for each module. Consistent with SDT, scripts emphasized satisfaction of the basic psychological needs of autonomy (choice and control), competence (knowledge, skill), and relatedness (connection to important others). This was done by providing families with choices as they navigated the modules, offering guidance regarding responses to common situations families encounter regarding T1D management, and emphasizing how to work together to manage blood glucose.

**Table 1:** Family Teamwork session topics.

Session	Topic
1	Diabetes and the family Challenges of diabetes
2	Tools Blood sugar monitoring Hemoglobin A_1c_
3	Checking blood sugars Talking about blood sugars Avoiding blame
4	Sharing the burden Identifying blood sugar patterns Individualizing care
5	Flexibility in meal planning Carbohydrate counting Exercise
6	Reassessment of the burden Preventing burnout Achieving flexibility
7	Miscarried helping Interdependence versus independence Reducing conflict
8	Review
9	Research and technology update Advances in monitoring devices

**Figure 3:**
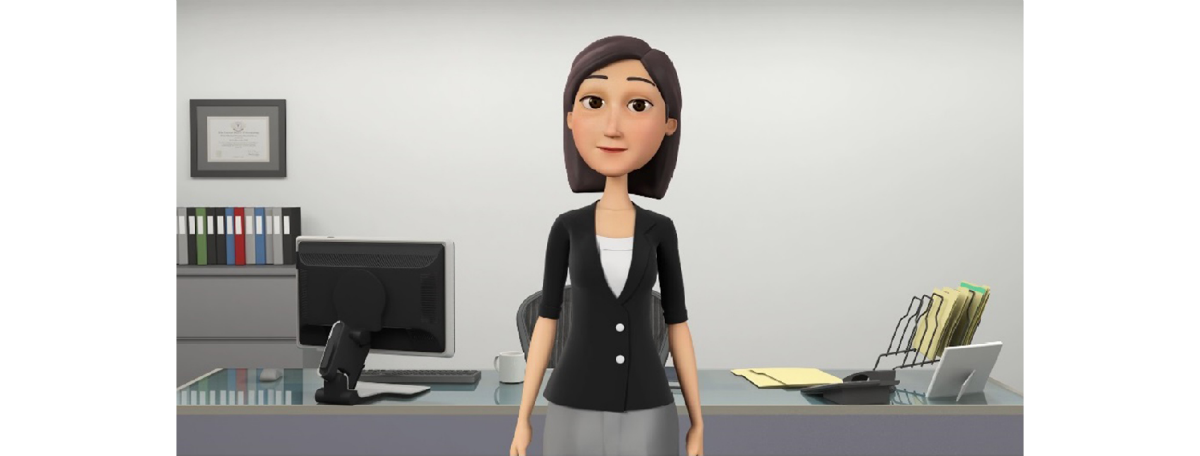
Image of Ashley, the relational agent in Diabetes Family Teamwork Online.

**Figure 4:**
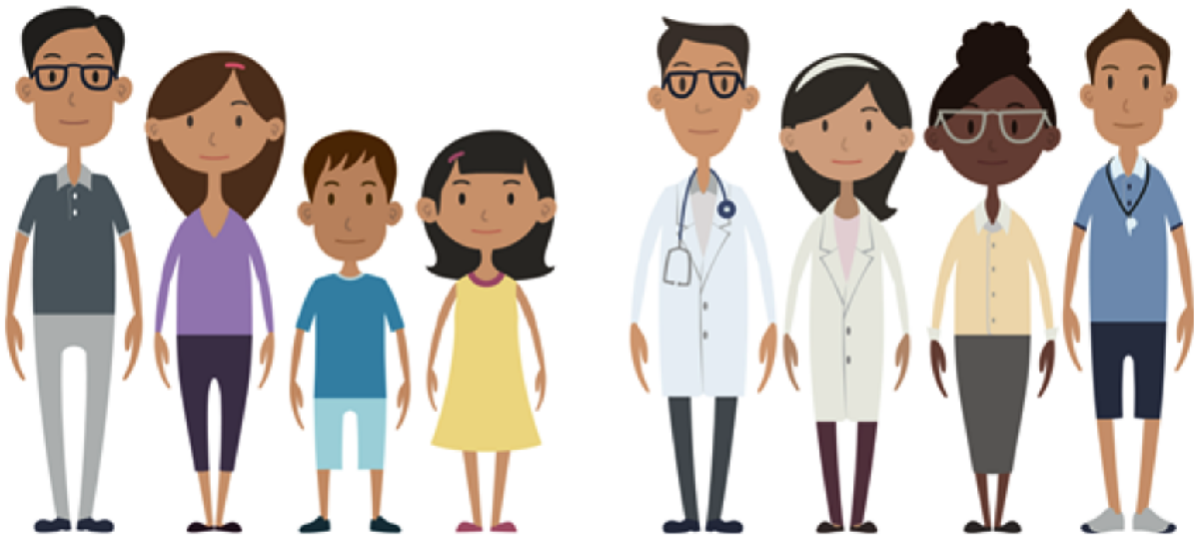
Cast of characters.

As part of the development work [34], parents and adolescents were interviewed separately to elicit their opinions about what the Ragt should look like, including appearance, sex, age, and clothing. They were also asked for suggestions regarding names for the Ragt. Using this information as a guide, a female Ragt named Ashley, with a professional, yet approachable, appearance was created (Figure 3). She was animated and designed to mimic in-person delivery by a human diabetes care provider. Prerecorded vocal segments by a professional voice actor were synced to Ashley’s character, enabling her to voice the scripted segments and convey emotion, concern, and respect for the families through vocalizations, facial expressions, and body movements. Consistent with past feedback, Ashley was designed to be “exotic” (ie, she was designed so that she appeared to be of no particular race or ethnicity), thus enabling families to assign their preferred race/ethnicity to her. In addition to Ashley, there were eight other characters—a family, consisting of a father, mother, son, and daughter, and four adult characters, consisting of a male and female physician, a teacher, and a coach (Figure 4).

Each module lasted approximately 20 minutes and focused on a different topic relevant to family communication around diabetes management (Figure 2). Guided by the flow diagram (Figure 1), modules were delivered in a set format that included topic introduction by Ashley; typical family scenarios common to a particular problem or issue relevant to the topic of a particular module; animatics, where typical family reactions (ranging from ideal to less than ideal) to common parent/adolescent situations arising around T1D were portrayed; and interactive components, where Ashley posed questions and the parent/adolescent dyad selected responses from a preselected list of responses, followed by a summary delivered by Ashley regarding the question or scenario. Each module ended with a joint parent/adolescent goal-setting task related to the module topic and family communication around diabetes management. Families were provided with several goals from which they could select. They could then print their goal and a tip sheet. At the beginning of the next module, the family reported goal attainment and received feedback from Ashley. Following completion of the module, they also received an email with the goal they selected and the tip sheet to minimize potential problems associated with not having ready access to a printer.

The program was designed to be viewed over a high-speed internet connection on a variety of devices (eg, desktop computer, laptop, mobile device). It was hosted over a secure, password-protected website. Parents and adolescents received separate passwords with which to log on and view the module. For the initial viewing of module, parents and adolescents were required to log on together. After completing the initial viewing, parents and adolescents could log on separately to view previously completed modules unlimited times. Families were eligible to view a new module and set a new goal 2 weeks after completing the previous module. If a family logged on before they were eligible to view the next module, they had access to previously completed modules only. Families were given approximately 3 months to complete the program.

A secure, password-protected database associated with FTO recorded each family’s information as they navigated the program, including log-ons, responses, and module completion. The researcher accessed the database through a secure dashboard that enabled them to view each family’s progress through the program including log-ons, responses, and goals. After completion of baseline data collection, the intervention coordinator entered the family into the database and assigned passwords. To protect confidentiality, each family received a unique study identifier, and this identifier, rather than their actual names, was entered into the database. A master list of actual names, identifiers, and passwords was stored on a secure, password-protected server available only to the study team.

### Recruitment

Families were recruited from a diabetes clinic at a large, tertiary care hospital for children. The study coordinator identified eligible families using the upcoming clinic schedule and the electronic medical record. Inclusionary criteria for the adolescents included being a current patient, aged 10 to 15 years old, diagnosed with T1D for at least 1 year, with English fluency, having high speed internet access, staying in the area for the duration of the study, and having a parent or legal guardian willing to participate in the study. Exclusionary criteria for the adolescents included having an average HbA_1c_ over the past year greater than 12% or less than 7%, inability to attend regular clinic visits, unwillingness to have interviews audio recorded, or having a physical or mental disease or condition that would conflict with study activities.

Inclusionary criteria for the parents included being the primary diabetes caregiver of the adolescent participating in the study, willingness to participate in study activities, with English fluency, having access to high speed internet, and staying in the area for the duration of the study. Exclusionary criteria included inability to attend regular clinic visits, unwillingness to have interviews audio recorded, or having a physical or mental disease or condition that would conflict with study activities.

Eligible families were sent a letter and flyer in the mail notifying parents that a study coordinator would meet the family at their upcoming clinic visit to speak about the study. Prior to the clinic visit, a study coordinator contacted the parent, informed them about the study, and if the parent was interested, screened for eligibility. If the parent was not available by phone, a study coordinator met the family at their clinic visit, described the study, and if the family was interested, screened for eligibility. Parents provided written informed parental consent and each child assented prior to study participation. The institutional review board at Baylor College of Medicine approved the protocol (H-37245).

### Intervention Procedures

After completing baseline data collection, the intervention coordinator sent the parent and adolescent separate emails with the website link, username, and private password. Families were provided with a program guide with instructions on how to use the website, including how to log on, how to view the modules, how to click the Back and Next buttons, and how to print the goal and tip sheets.

After the family completed a module, the intervention coordinator sent a thank you email to the parent’s email address with the available date for the next module and a reminder that the family could log on between modules and view the previously completed modules. The goal (set by the family) and tip sheets for the module were also attached to the thank you email. The intervention coordinator kept track of the families logging on to the modules. If families were not logging on regularly to watch the next eligible module, the coordinator sent reminder emails to both the parent and adolescent and followed up with a phone call after the initial reminder email.

### Data Collection Procedures

Several types of data were collected: self-report data at baseline, post 1 (immediately after completing the intervention), and post 2 (3 months after post 1); interviews (post 1, post 2); intervention (ie, log-ons, in-module responses); and staff logs (Table 2). Parents and adolescents each received monetary incentives for completing surveys and/or interviews at each data collection time point ($50 baseline, $70 post 1, $80 post 2). No incentives were provided for completing intervention modules.

**Table 2:** Data sources.

Type	Who	Method	Recruitment	Baseline	Intervention	Post 1	Post 2
Self-report	Parent; adolescent	Online		✓		✓	✓
Interviews	Parent; adolescent	Telephone				✓	✓
Module	Family	Backend database			✓		
Logs	Staff	Tracking system	✓	✓	✓	✓	✓

### Feasibility Criteria

A priori feasibility criteria were established to guide the determination of whether FTO was feasible and acceptable [34]. Minor adjustments were made to the criteria to account for differences in measures or scoring approaches. Final criteria used to assess feasibility were (1) recruitment goals were met (n=20), (2) families completed ≥75% of the modules, (3) attrition rate was ≤10%, (4) program satisfaction was high (≥80% of families), (5) therapeutic alliance with the Ragt was high (average score of ≥60/84), (6) families expressed positive attitudes toward the Ragt (average item score of ≥5 on ≥4 items), (7) ≥80% of data were collected at post 1 and post 2, and (8) few technical issues (<10%) occurred during intervention delivery.

### Measures

Recruitment was assessed with logs maintained by the research staff (eg, number of eligible families with clinic visits in the recruitment period, number of eligible families who enrolled and reasons, number of eligible families who did not enroll and reasons). The recruitment goal was 20 families. This criterion was assessed by comparing the number of eligible families who enrolled to the criterion.

Module completion was defined as parent and adolescent logging on to the program website at the same time to complete the module activities. This information was automatically collected when families logged on and interacted with the program. Module completion was calculated by dividing the total number of modules completed by the family by the total number of available modules.

Attrition rate was defined as the number of families who did not complete the intervention (ie, all 4 modules) and all data collection activities (ie, baseline, post 1, post 2). It was calculated by dividing the total number of families who did not complete all these activities by the total number of families who enrolled in the study.

Program satisfaction was assessed among parents and adolescents at post 1 with a 10-item measure of satisfaction used in previous studies [30,35,36]. Items were rated using a 3-point Likert scale (yes, not sure, no). Responses were summed to determine overall program satisfaction. Scores could range from 10-30, with lower scores representing higher satisfaction. Separate scores were calculated for parents and adolescents.

Therapeutic alliance was assessed among parents and adolescents at post 1 with the Bond subscale of the Working Alliance Inventory, a 12-item measure assessing trust and belief the participant can work with their provider (ie, Ragt) to achieve the desired outcomes [37]. Items were rated on a 7-point Likert scale, ranging from always hard to always easy. For 3 items, the desired response was never instead of always; therefore, these items were reverse coded, with never being the desired response. Responses were summed to create a total scale score. The total score could range from 12 to 84, with higher scores representing greater therapeutic alliance. Separate scores were computed for parents and adolescents.

Attitudes toward the Ragt were assessed among parents and adolescents at post 1 with a 6-item survey adapted from Bickmore et al [27]. Attitudes were assessed with items addressing different dimensions of the Ragt, including usability, continuance, relationship, preference, adherence, and satisfaction [27]. Items were rated on a 7-point scale, ranging from less desirable to more desirable attitudes (Table 3). Response scales differed for items; therefore, responses were scored and reported individually. Higher scores represented more positive attitudes.

Data completeness was defined as the percentage of families with complete self-report data at all assessment points (baseline, post 1, post 2).

Technical issues were defined as the number of incidents reported by families that limited access to the program and were determined from a review of staff logs.

**Table 3:** Attitude scale (adapted from Bickmore et al [27]).

Attitude	Questions	Response anchors
Usability	How easy was it to “talk” with Ashley?	1 (always hard) to 7 (always easy)
Continuance	How much would you like to continue working with Ashley?	1 (not at all) to 7 (very much would like)
Relationship	How would you describe your relationship with Ashley?	1 (complete stranger) to 7 (always a friend)
Preference	Would you have rather talked with your doctor or nurse rather than Ashley?	1 (definitely prefer doctor or nurse) to 7 (definitely prefer Ashley)
Adherence	How likely is it that you will follow Ashley’s advice?	1 (very unlikely) to 7 (very likely)
Satisfaction	How satisfied were you with Ashley?	1 (very dissatisfied) to 7 (very satisfied)

To obtain an understanding of parent and adolescent perceptions toward the intervention, telephone interviews were conducted following completion of post 1 and post 2 data collection surveys. The purpose of the post 1 interview was to identify perceptions related to the intervention, while the post 2 interview explored perceptions related to maintenance. Only post 1 interviews will be reported here to provide a deeper understanding of program feasibility and acceptability from the perspective of the adolescents and parents who participated in the study. Interviews were scripted and conducted by trained interviewers. Probes and prompts were used as needed to expand, clarify, and understand responses.

### Statistical Analyses

Descriptive statistics were calculated on survey data. Separate analyses were calculated for parents and adolescents. Results were compared to the criteria to make a determination of feasibility. Audio recordings of interviews were reviewed and key point summaries created to capture important thoughts that emerged from adolescent and parent interviews to enhance understanding of results [38]. Verbatim quotes were used to provide voice to adolescent and parent perceptions and experiences.

## Results

### Participants

Parents were mostly female (25/26, 96%), white (21/26, 81%), non-Hispanic (22/26, 85%), and married or living with significant other (22/26, 85%). Families had college-level education or higher (20/26, 77%) and reported household incomes of greater than $61,000 (20/26, 80%; Table 4).Adolescents were mostly female (19/26, 73%), white (22/26, 85%), and non-Hispanic (21/26, 81%). There were slightly more adolescents aged 13 to 15 years in the study (14/26, 54%). Disease duration ranged from 2 (4/26, 15%) to 13 years (1/26, 4%; Table 5).

**Table 4:** Descriptive statistics of parents who completed the Diabetes Family Teamwork Online study (n=26).

Characteristic		Value, n (%)	
**Gender**			
	Male		1 (4)
	Female		25 (96)
**Race/ethnicity**			
	American Indian/Alaskan Native		1 (4)
	Black		3 (12)
	White		21 (81)
	Other		1 (4)
**Hispanic**			
	Hispanic		4 (15)
	Non-Hispanic		22 (85)
**Marital status**			
	Married/living with significant other		22 (85)
	Single, never married		2 (3)
	Divorced, separated, or widowed		1 (4)
	Other		1 (4)
**Highest household education**			
	High school graduate or equivalent		3 (12)
	Technical school		1 (4)
	Some college		2 (8)
	College graduate		11 (42)
	Postgraduate study		9 (35)
**Household income^a^**			
	$21,000-$41,000		3 (12)
	$42,000-$61,000		2 (8)
	Greater than $61,000		20 (80)

^a^One parent did not report household income.

**Table 5:** Descriptive statistics of adolescents who completed the Diabetes Family Teamwork Online study (n=26).

Characteristic		Value, n (%)	
**Gender**			
	Male		7 (27)
	Female		19 (73)
**Race/ethnicity**			
	American Indian/Alaskan Native		1 (4)^a^
	Black		3 (12)
	White		22 (85)
**Hispanic**			
	Hispanic		5 (19)
	Non-Hispanic		21 (81)
**Age (years)**			
	10-12		12 (46)
	13-15		14 (54)
**Disease duration (years)**			
	2		4 (15)
	3		8 (31)
	4		2 (8)
	5		3 (12)
	6		3 (12)
	7		1 (4)
	8		2 (8)
	10		1 (4)
	12		1 (4)
	13		1 (4)

^a^Participant also identifies as white.

### Feasibility Outcomes

Feasibility criteria and status are summarized in Table 6. Feasibility of each criterion is briefly described below.

**Figure 5:**
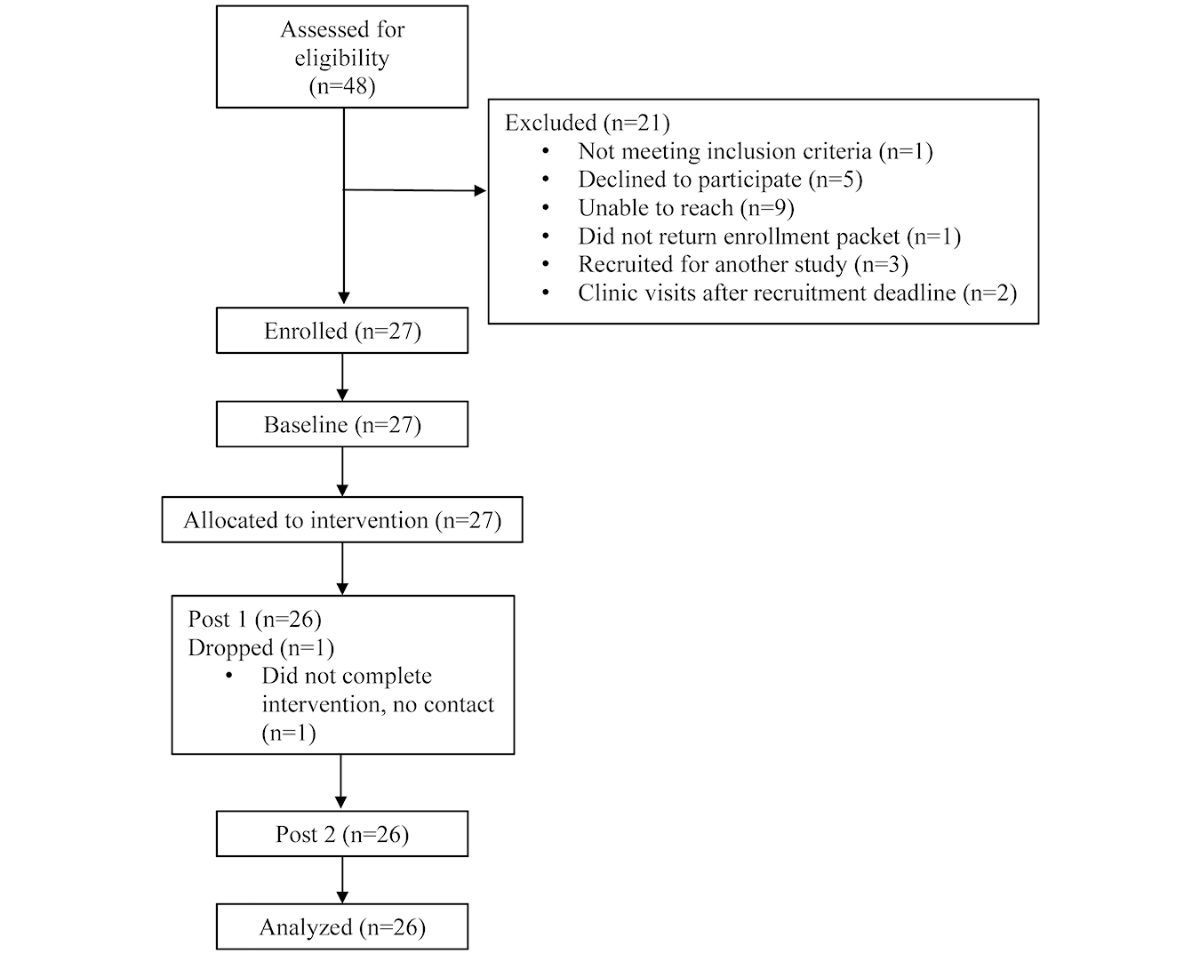
Family Teamwork Online CONSORT (Consolidated Standards of Reporting Trials) diagram.

Recruitment began in October 2016 and ended in December 2016. A review of clinic records indicated that 48 families met general eligibility criteria and were notified of the study and their eligibility to participate. Of these, 5 declined to participate, 9 could not be contacted, 3 were enrolled in another study that made them ineligible for our study, and 4 were not included for reasons ranging from clinic visit scheduled after recruitment closed (2), not meeting inclusionary criteria (1), and not returning recruitment packet (1). Of the eligible families, 27 enrolled, completed baseline data collection, and were allocated to the intervention (Figure 5). Our recruitment goal was 20 families; therefore, this criterion was exceeded.

FTO consisted of 4 modules. All 4 modules were completed by 26 out of 27 families enrolled in this study. The feasibility criterion was >75%; therefore, this criterion was met.

Of the 27 families who enrolled in the study, 26 completed the intervention and all data collection (baseline, post 1, post 2) and intervention activities. The feasibility criterion was ≤10%; therefore, this criterion was met.

The mean program satisfaction scores were 11.08 (SD 1.32) for adolescents and 10.46 (SD 0.65) for parents, indicating that over 90% of adolescents and parents reported high program satisfaction. The feasibility criterion was ≥80% of adolescents and parents reported high program satisfaction. Therefore, this criterion was met, with both groups reporting high program satisfaction.

The mean therapeutic alliance score for adolescents was 64.58 (SD 14.43) and 64.65 (SD 14.33) for parents. The feasibility criterion was an average total scale score of ≥60/84. Therefore, this criterion was met for both adolescents and parents.

**Table 6:** Feasibility criteria.

Criterion	Standard	Status
Recruitment	20 families	Exceeded
Module completion	≥75% modules	Met
Attrition rate	≤10%	Met
Program satisfaction	≥80%	Met
Therapeutic alliance	Average score of ≥60/84	Met
Attitudes toward Ragt	Score of ≥5 on ≥4 items	Met
Data completeness	≥80%	Met
Technical issues	≤10%	Met

Both adolescents and parents reported attitude scores toward Ashley of at least 5 on 5 of the 6 attitude items, respectively: usability (5.85 [SD 1.43], 5.50 [SD 1.39]), continuance (5.42 [SD 1.17], 5.23 [SD 1.03]), relationship (5.42 [SD 1.55], 5.08 [SD 1.52]), preference (2.73 [SD 1.59], 3.54 [SD 1.49]), adherence (5.73 [SD 1.12], 6.00 [SD 0.69]), and satisfaction (6.0 [SD 1.02], 5.96 [SD 0.72]). Therefore, this criterion was met.

Twenty-six of the 27 families had complete self-report data at all assessment points. The feasibility criterion was ≥80%; therefore, this criterion was met.

No technical issues were reported by the families. The feasibility criterion was “few technical issues”, defined as <10%. Therefore, this criterion was met.

The qualitative findings generally supported the survey results. Most adolescents and parents liked the program. They generally thought the content was appropriate, easy to understand, and realistic.

Well, I thought it was useful in what we deal with every day—high blood sugars or the low blood sugars or what we need to be doing better when it comes to managing [daughter’s name] diabetes. The examples were pretty common to what we deal with, so it helped to see it being played out for us and how families responded and if our response was appropriate for that situation. I thought it was helpful in helping us understand how to react with the high blood sugar or low blood sugar or just certain situations we deal with every day.

Parent

They thought that the module number and length were appropriate.

I think they were just about right. I mean, I liked them as they were. I wouldn’t have liked them to be longer or shorter.

Adolescent

This sentiment seemed to be echoed by parents.

I liked the time. I didn’t think they were too long or too short ‘cause it was so detailed with every session, but it didn’t take a lot of my time or my child’s.

Parent

A few families wanted longer or more modules on certain topics. For example, a parent suggested adding information on tricky topics.

I think four is okay. I think for some of the more tricky topics, maybe adding a follow-up for that that goes into more detail might be helpful.

Parent

A adolescent expressed also wanting more information.

I think it was too little...6 or 7...cause I wanted to learn more about what they were talking about.

Adolescent

Parents and adolescents also seemed to like the family focus taken by the program.

I liked it. It was pretty fun experiencing it with my mom and how we got to like work out my diabetes better. It was pretty fun.

Adolescent

Overall, parents shared positive feedback on the Ragt, including her appearance, responses, and voice.

I thought she was very helpful. She was very helpful in answering things, and like for something quick, like a quick question or whatever, I mean her response to things was better than having a doctor right there in your face. She was professional. She was child friendly and adult friendly.

Parent

Adolescents had similar reactions.

I think she was helpful, and I think that—I don’t know if it’s just me—since she’s a girl, it’s easier to listen to someone the same as me.

Adolescent

A few adolescents expressed that although they liked Ashley, she had limitations.

I think she was fine, but I would rather have an actual person...because Ashley didn’t really give that much detail.

Adolescent

Families were also asked what information in the program was most helpful or useful. Parents shared a variety of information that was most helpful for them, including the information presented on parent reactions to high and low blood sugars, not referring to blood sugars as good and bad, communication between the parent and adolescent, setting goals, and diabetes burnout. Two parent quotes eloquently summarize parent thoughts regarding what they perceived as most helpful:

Well for me it would be the reminder to let the teen take charge and let the parent sort of step back and say what would you do differently instead of managing it myself.

Parent

That [diabetes burnout] was probably the best one for us because we really didn’t know a lot about that.

Parent

Adolescents had similar reactions and identified a variety of topics that were helpful. They seemed to particularly focus on issues related to family communication around diabetes management.

How to control like no blaming and everyone’s part of the team.

Adolescent

The focus on using different language was also viewed as important.

The most helpful activity was the one where you had to use different words instead of it was like a bad or good blood sugar, it was a high or a low.

Adolescent

When asked what was least helpful in the program, few parents or adolescents had many suggestions to offer. Most thought the program was helpful.

I don’t really think there was just anything so unhelpful that I would leave it out.

Parent

This sentiment was echoed by adolescents.

I don’t know. I don’t think anything was bad about the program. I liked it myself, so I don’t think anything was least helpful. Everything was pretty helpful to me.

Adolescent

A few suggestions were offered, however.

For me, it was all a good reminder, but the probably least helpful was looking at the numbers as good or bad. I always try to look at them as information...that was probably the least helpful because I kind of already look at it that way.

Parent

Taking the snack and checking your blood sugars and stuff like that when you’re exercising.

Adolescent

## Discussion

### Principal Findings

This paper reports on the feasibility of delivering a program to enhance communication around blood glucose management tasks in families with an adolescent who has T1D. Feasibility criteria were met or exceeded. Therefore, we concluded this program was a feasible method for promoting enhanced communication around diabetes management tasks.

Recruitment goals for this study were exceeded in a relatively short period of time. Conflict around diabetes management tasks in families with adolescents who have T1D and its association with poorer diabetes outcomes have been well documented in the literature [9,39,40]. There is also clear evidence that both parents and adolescents perceive there is conflict around diabetes management [41,42]. The short time period within which recruitment goals were exceeded suggests that parents and adolescents are interested in learning to manage or reduce this conflict.

Participation by families was high, as indicated by the low attrition rate and high program completion rates. This suggests that families found the program helpful and a beneficial use of their time. This is not surprising, however, given the success of FT, the program on which FTO was based [9,12,13]. This finding also adds to the body of literature supporting communication technologies as a potentially effective way to extend the reach of diabetes management programs for adolescents [16]. Future research is needed to assess the efficacy of this approach.

Therapeutic alliance is the emotional dimension associated with working with a provider and their perceived ability to help the patient achieve a desired outcome [37]. Although parents and adolescents were aware that Ashley was a digital image and not an actual person, therapeutic alliance was established. This was similar to results reported by Bickmore et al [43] when a Ragt was used to encourage exercise in a healthy population and deliver hospital discharge instructions to patients with depressive symptoms [27]. Given the differences between initiating a healthy behavior such as exercise and receiving instructions for posthospital care versus potential consequences of poor glycemic control [44], this suggests that Ragts may be an acceptable way to extend care for a variety of diverse behaviors outside of traditional settings. In the management of a chronic condition, such as T1D, patient-provider communication is an essential component of care [45]. Therefore, our goal was not to supplant this relationship but to extend care outside the clinical setting. Although this study suggests Ragts are a feasible and acceptable method of achieving this goal, future research is needed to examine ways in which Ragts may further extend the patient-provider relationship outside the clinical setting. One way to achieve this may be to design Ragts that respond emphatically in real time, based on the participant’s expressed emotions [46]. Further, as suggested by the qualitative results, it may be that therapeutic alliance takes on a different form with a Ragt. Future research is needed to more fully investigate this possibility.

Both parents and adolescents reported positive attitudes regarding ease of use, desire to continue working with Ashley, their relationship with Ashley, the likelihood of adhering to her advice, and satisfaction with her. The exception was their stronger preference for talking with their doctor or nurse, rather than Ashley, about T1D. As discussed above, given that the patient-provider relationship is essential to the care of T1D, particularly in adolescents [45], this finding is not surprising. The goal when working with chronic disease management should not be to supplant or interfere with the patient-provider relationship but to augment and support it and extend care beyond the clinical setting. Given that this is the first study to use Ragts to enhance communication around blood glucose management tasks in families with an adolescent who has T1D, it adds important information to the literature regarding expectations around chronic disease management and ways in which to augment and extend care between visits with the health care provider. Future research with providers may provide insight into ways Ragts could be used to further extend care into the home environment.

Although missing data are a common problem in research that can present challenges in the analytic stage [47], we had complete data on all but one family. Consistent with guidelines developed to minimize missing data [48], data collection was online, making participation convenient, and the families received notifications when they were eligible to participate in data collection activities. We also ensured contact information for each family was current, study coordinators were experienced and well trained in study procedures, participation in data collection activities was monitored and tracked, and incentives were provided to the families for completing data collection at each assessment point. All of these activities likely contributed to the high data collection rates.

Finally, families reported no technical issues accessing or using the program to the intervention team. This is likely due to working with a highly skilled local Web design company with whom we have a history of successful collaboration. Further, experience has taught us the importance of knowing in advance how the individual pieces of the intervention fit together, as well as the overall program flow. A template was created for the modules, and this template guided each module. This consistency may have contributed to fewer technical glitches. A program guide was also created and shared with families prior to their viewing of the first module, which may have further contributed to fewer technical issues. Because technical issues can be annoying and may disrupt program participation, particularly when they occur multiple times or at critical points in program delivery, the lack of technical issues may have also contributed to our high program participation and satisfaction ratings.

### Limitations

Although this study has strengths, including being an adaptation of an intervention previously found to be efficacious and its theoretical grounding, it is not without limitations. The small sample size and one-group design limits its ability to find statistical significance in key outcome variables, such as family conflict and communication around blood glucose. However, this was designed to be a feasibility study, with the goals of assessing the viability of this approach, needed changes, and whether it should move forward for an assessment of efficacy with a larger, more fully powered randomized controlled trial [49]. Parents and adolescents in the study were predominantly female and the Ragt was female, which may have influenced the appeal of this approach to families who participated in the study. However, the appearance, sex, and age of the Ragt designed for this study was informed by formative research with parents and adolescents other than those who participated in the feasibility study; this suggests the Ragt may have broad appeal regardless of sex. This study was also conducted in a limited geographic region with a sample of families who were predominately white, of higher socioeconomic status, and well educated, limiting the ability to draw conclusions about the generalizability of this approach with other populations. Finally, because this study uses technology, it may not be appropriate for use in locations that may have limitations regarding internet access or speed, such as some rural areas, and it may have broader appeal among families with adolescents who use technology to facilitate blood glucose management (eg, continuous glucose monitoring, insulin pumps).

### Conclusion

An online intervention to enhance communication around blood glucose management task in families with an adolescent who has diabetes was found to be feasible and acceptable. The contribution of this research to the literature is that it demonstrates that Ragts are a feasible adjunct to traditional care and may be a way to extend care outside the clinical setting. Future research is needed to identify the effects on family communication, quality of life, blood glucose, and diabetes management in a larger, more fully powered sample.

## Abbreviations

CGMS: continuous glucose monitoring system
DCCT: Diabetes Control and Complications Trial
FT: Family Teamwork
FTO: Diabetes Family Teamwork Online
HbA_1c_: hemoglobin A_1c_
Ragt: relational agent
SDT: self-determination theory
T1D: type 1 diabetes
USDA/ARS: US Department of Agriculture/Agricultural Research Service
